# Language Use in Chinese University Students With Depressive Symptoms

**DOI:** 10.1002/pchj.70041

**Published:** 2025-07-22

**Authors:** Nantong Wang, Ruichao Zhou, Chenyang Liu, Xiaolu Zhou, Changlai Chen, Raymond C.K. Chan

**Affiliations:** ^1^ School of Education Shanghai Normal University Shanghai China; ^2^ School of Statistics and Mathematics Shanghai Lixin University of Accounting and Finance Shanghai China; ^3^ International College of Chinese Studies, Shanghai Normal University Shanghai China; ^4^ Neuropsychology and Applied Cognitive Neuroscience Laboratory, State Key Laboratory of Cognitive Science and Mental Health Institute of Psychology, Chinese Academy of Science Beijing China; ^5^ Department of Psychology University of Chinese Academy of Sciences Beijing China

**Keywords:** Chinese university students, depression, language use

## Abstract

This study examined the language use in Chinese university students with depressive symptoms based on negative and positive memory recall tasks. People with depression used more first‐person singular pronouns in the negative memory task and more negative words in both memory tasks.

Language use reveals mental health status. Spurred by the advances of computerized text analysis, accumulating studies have examined the associations between language use, or more specifically, word use and mental health conditions. Several western studies (e.g., Rude et al. 2004) identified the use of first‐person singular pronouns, negative, and positive words as the meaningful marker of depression, despite some conflicting results. At present, assessment of depression is still mostly relying on reports of clients as well as experiences of practitioners, which are easily biased by subjectivity. Observing people's word choice is considered to be a potential new screening measure for depression that is objective, non‐intrusive, and easy to obtain. However, it is unclear whether we can apply the same language‐based assessment of depression to Chinese settings.

Pyszczynski and Greenberg (1987) proposed that depression involves excessive self‐focus, after loss of important sources of self‐worth, such as a job. This maladaptive self‐focus produces intensified negative affect and mounted self‐criticism and self‐blame, which maintain and exacerbate depression. The more use of the first‐person singular pronoun was identified as a marker of the psychopathology of self‐focused characterized depression. Notably, Pyszczynski and Greenberg (1987) found that depressive self‐focus style occurred in the context of negative events but not positive events. However, language contexts have often been ignored in research on language use studies of depression. We speculated that the more use of first‐person singular pronouns similarly occurs in negative but not positive narrations.

Beck (1967) described the characteristic cognition of depression as a negative triad: a negatively biased view of the self, future, and world. People with depression were found to use more negative words than healthy controls (HCs) (e.g., Rude et al. 2004). Moreover, people with depression were found to express fewer positive words compared to healthy individuals (e.g., Rude et al. 2004). Do people with depression use more negative words and fewer positive words, irrespective of language contexts? The extant literature provides mixed findings.

The extant literature suggests that the use of first‐person singular pronouns reflects self‐focus in both East and West (Chung and Pennebaker 2007), and emotion words show similar valence and activation properties across different languages (Jackson et al. 2019). Therefore, this study aimed to examine word use (i.e., first‐person singular pronouns, negative and positive words) in Chinese university students with depressive symptoms, based on the positive and negative autobiographical memory recall tasks. We hypothesized that university students with depressive symptoms would use more first‐person singular pronouns in the negative memory recall task but not the positive memory task; more negative words and fewer positive words irrespective of the valence of tasks.

Sixty‐one students (19–25 years old) were recruited from several universities in Shanghai. Thirty participants (*M*_age_ = 22.77 years, SD = 1.96, 8 males) reporting scores above 19 on the Center for Epidemiological Studies Depression Scale (CES‐D, Zhang 1998) were classified as people with depressive symptoms (DS). The remaining 31 participants (*M*_age_ = 22.65 years, SD = 1.58, 9 males) reporting scores below 16 were classified as HCs. The experiment consisted of positive and negative autobiographical memory recall tasks (Rottenberg et al. 2006). Each task lasted for 6 min and was presented in random order on a computer screen. Participants were instructed to describe the saddest event they had experienced in the negative memory recall task and the happiest event in the positive memory recall task. Participants' reports were analyzed by a Chinese text analysis program TextMind (Gao et al. 2013) that calculated the frequencies of first‐person singular pronouns/negative words/positive words out of the total words in each task.

Three 2 × 2 mixed model ANOVAs were conducted, with Group (DS, HC) as the between‐subjects variable, Context (happy, sad) as the within‐subjects variable, and the use of first‐person singular pronouns, negative and positive words as dependents. Regarding the use of first‐person singular pronouns, the Group main effect was significant (*F*_(1,59)_ = 4.076, *p* = 0.048, *η*_*p*_^2^ = 0.065). Follow‐up comparisons suggested that the DS group used more first‐person singular pronouns in the sad memory recall task (*M* = 0.057, SD = 0.026) than the HC group (*M* = 0.041, SD = 0.020, *F*_(1,59)_ = 7.537, *p* = 0.008, *η*_*p*_^2^ = 0.113). The main effect of Context and the interaction of Group × Context both failed to reach significance (*p*s > 0.05).

Regarding the use of negative words, the Group main effect was significant (*F*_(1,59)_ = 8.894, *p* = 0.004, *η*_*p*_^2^ = 0.131). Followed‐up comparisons indicated that the DS group used more negative words not only in the sad memory recall task (*M* = 0.020, SD = 0.009) than the HC group (*M* = 0.015, SD = 0.009, *F*_(1,59)_ = 4.094, *p* = 0.048, *η*_*p*_^2^ = 0.065), but also in the happy memory recall task (*M* = 0.015, SD = 0.014) than the HC group (*M* = 0.009, SD = 0.007, *F*_(1,59)_ = 5.320, *p* = 0.025, *η*_*p*_^2^ = 0.083). The main effect of Context was significant (*F*_(1,59)_ = 9.908, *p* = 0.003, *η*_*p*_^2^ = 0.144); participants used more negative words in the sad memory recall task (*M* = 0.018, SD = 0.009) than in the happy memory recall task (*M* = 0.012, SD = 0.012). The interaction of Group × Context failed to reach significance (*p* > 0.05).

Regarding the use of positive words, the main effect of Context was significant (*F*_(1,59)_ = 134.832, *p* < 0.001, *η*_*p*_^2^ = 0.696); participants used more positive words in the happy memory recall task (*M* = 0.040, SD = 0.015) than in the sad memory recall task (*M* = 0.010, SD = 0.010). The Group main effect and interaction of Group × Context both failed to reach significance (*p*s > 0.05).

The present findings suggested the suitability of a language‐based assessment of depression applied to Chinese university students. Chinese students with depressive symptoms differed from healthy individuals in the use of first‐person singular pronouns and negative words, but not in the use of positive words. These findings were partially consistent with findings of western studies (e.g., Rude et al. 2004). We found no group difference in the use of positive words between Chinese people with and without depressive symptoms, using traditional ANOVA. However, a subsequent Bayesian mixed model ANOVA provided strong evidence supporting the interaction of Group × Context (BF_10_ = 5.240 × 10^18^) in the use of positive words. Future studies should recruit a large sample in the Chinese settings.

The main contribution of the present study suggests that word uses may be differently moderated by language contexts. People with depressive symptoms only used more first‐person singular pronouns in negative narrations. This finding supports the theoretical claims that the depressive self‐focus style is present in negative events rather than positive events (Pyszczynski and Greenberg 1987). However, people with depressive symptoms may use more negative words than non‐depressed people irrespective of the valence of the language contexts.

## Ethics Statement

This study was approved by the ethics committees of Shanghai Normal University (SHNU [2021] protocol number: 16).

## Consent

We obtained written consents from all participants.

## Conflicts of Interest

The authors declare no conflicts of interest.

## Data Availability

The data that support the findings of this study are available from the corresponding author upon reasonable request.
